# Nanozyme‐Catalyzed Metasurface Plasmon Sensor‐Based Portable Ultrasensitive Optical Quantification Platform for Cancer Biomarker Screening

**DOI:** 10.1002/advs.202301658

**Published:** 2023-06-26

**Authors:** Rui Li, Hongli Fan, Hanlin Zhou, Youqian Chen, Qingcai Yu, Wenjun Hu, Gang L. Liu, Liping Huang

**Affiliations:** ^1^ College of Life Science and Technology Huazhong University of Science and Technology 1037 Luo Yu Road Wuhan 430074 P. R. China; ^2^ Biosensor R&D Department Liangzhun (Wuhan) Life Technology Co., Ltd. 666 Gaoxin Avenue Wuhan 430070 P. R. China; ^3^ School of Life and Health Science Anhui Science and Technology University Fengyang 233100 P. R. China

**Keywords:** artificial nanozyme, cancer biomarker, enzyme‐linked immunosorbent assay, metasurface plasmon sensor, surface plasmon resonance biosensor

## Abstract

Developing plasmonic biosensors that are low‐cost, portable, and relatively simple to operate remains challenging. Herein, a novel metasurface plasmon‐etch immunosensor is described, namely a nanozyme‐linked immunosorbent surface plasmon resonance biosensor, for the ultrasensitive and specific detection of cancer biomarkers. Gold‐silver composite nano cup array metasurface plasmon resonance chip and artificial nanozyme‐labeled antibody are used in two‐way sandwich analyte detection. Changes in the biosensor's absorption spectrum are measured before and after chip surface etching, which can be applied to immunoassays without requiring separation or amplification. The device achieved a limit of alpha‐fetoprotein (AFP) detection < 21.74 fM, three orders of magnitude lower than that of commercial enzyme‐linked immunosorbent assay kits. Additionally, carcinoembryonic antigen (CEA) and carbohydrate antigen 125 (CA125) are used for quantitative detection to verify the universality of the platform. More importantly, the accuracy of the platform is verified using 60 clinical samples; compared with the hospital results, the three biomarkers achieve high sensitivity (CEA: 95.7%; CA125: 90.9%; AFP: 86.7%) and specificity (CEA: 97.3%; CA125: 93.9%; AFP: 97.8%). Due to its rapidity, ease of use, and high throughput, the platform has the potential for high‐throughput rapid detection to facilitate cancer screening or early diagnostic testing in biosensing.

## Graphical abstract

A nanozyme‐linked immunosorbent surface plasmon resonance biosensor integrating the low‐cost nano cup chip with the Au@Pt nanoflowers is developed, which can be used for an ultrasensitive (fM‐scale), rapid (minute‐level), and portable (pocket‐size) test for biomarker diagnosis. In order to verify the universality of the platform, three cancer biomarkers (alpha‐fetoprotein, carcinoembryonic antigen, carbohydrate antigen 125) are selected for quantitative detection. Most importantly, clinical samples are used to verify the accuracy and stability of the platform.

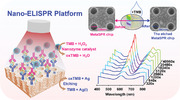

## 1 Introduction

Cancer is a non‐communicable disease responsible for the deaths of millions of people each year globally.^[^ ^1^ ^]^ In particular, liver cancer is the second most common cause of cancer‐related death globally, with morbidity and mortality rates accounting for 6% and 9% of the global cancer burden, respectively.^[^ ^2^ ^]^ Biomarkers have been used extensively for early cancer diagnosis to reduce incidence rates and cancer‐related deaths.^[^ ^3^ ^]^ For instance, increased serum levels of alpha‐fetoprotein (AFP)—a biomarker of primary liver cancer and hepatocellular carcinoma—may indicate liver cancer. Moreover, AFP reportedly regulates cancer development and angiogenesis in breast cancer, lymphoma cells, and hepatocellular carcinoma.^[^ ^4^ ^]^ However, healthy adults typically have extremely low AFP serum levels. As such, AFP has been developed as an effective biomarker for early liver cancer diagnosis.^[^ ^5^ ^]^ AFP expression levels exhibit significant differences among cancers; therefore, it is used as a positive detection index for various cancers. Additionally, a decline in AFP levels serves as a prognostic indicator, with a slow rate of decline implying less effective chemotherapy against malignancies.^[^ ^6^ ^]^ Therefore, non‐invasive and ultrasensitive biomarker detection devices are required to facilitate early diagnosis, which could improve cancer therapeutic interventions and patient outcomes.

Various strategies are commonly used to detect biomarkers (AFP as an example), including colorimetry,^[^ ^7^ ^]^ electrochemiluminescence,^[^ ^8^ ^]^ fluorescence,^[^ ^9^ ^]^ localized surface plasmon resonance (LSPR),^[^ ^10^ ^]^ and surface‐enhanced Raman spectroscopy.^[^ ^11^ ^]^ However, certain fundamental limitations of immunoassays, including severe fluorescence quenching and unsatisfactory sensitivity, hinder their application in clinical diagnosis. Moreover, the application of a single technique does not yield rapid results and is often limited in specificity or sensitivity, resulting in frequent false positive or false negative results. Hence, the development of a simple, low‐cost, rapid, and sensitive monitoring platform that does not require fluorescent labeling for the detection of cancer biomarkers to facilitate clinical diagnosis and screening, remains a compelling research goal.^[^ ^5^ ^]^

Although the lateral flow test is a representative point‐of‐care testing (POCT) platform and even quantitative detection is possible, its detection throughput and sensitivity are limited, consequently hindering its application in the large‐scale early screening of cancer markers.^[^ ^12^ ^]^ In addition, the most commonly used immunoassay method for clinical marker detection is enzyme‐linked immunosorbent assay (ELISA), due to its precision, specificity, low cost, and simple readout.^[^ ^13^ ^]^ However, during the early stages of certain diseases, the sensitivity of traditional ELISAs prevents the detection of ultra‐low biomarker concentrations. Moreover, ELISAs exhibit nonspecific binding due to enzyme instability.^[^ ^14^ ^]^ In contrast, surface plasmon resonance (SPR) and LSPR techniques have numerous advantages, including rapid high‐throughput, highly sensitive, non‐destructive analysis, as well as real‐time monitoring of binding status, which make them common modalities for the detection of cancer markers.^[^ ^15^ ^]^ However, the current methods may exhibit inadequate sensitivity or non‐specificity. Moreover, advanced signal amplification strategies are necessary to develop ultrasensitive, highly specific, and portable methods.

Metasurface plasmon resonance (MetaSPR) technology was initially developed for the portable and practicable stochastic biosensing of biomarkers. The MetaSPR biosensor is composed of a periodic uniform metal nanostructure array.^[^ ^16^ ^]^ A miniaturized light source shining on the grating structure of the sensor itself can produce collective oscillations of electronic gas and excite the reflected signal of the sensor without couplers or large precision instruments.^[^ ^17^ ^]^ Although the uniform nano cup structure endows the MetaSPR chip biosensor with unique optical characteristics, it has high associated costs and poor detection sensitivity. Therefore, it is necessary to create portable low‐cost biomarker detection instruments to implement POCT detection based on the MetaSPR principle, for both hospital and home requirements.

Artificial nanozymes based on nanomaterials with catalytic activities similar to enzymes are popular nano‐catalytic materials.^[^ ^18^ ^]^ Their catalytic performance is less affected by external environmental conditions, they exhibit stable chemical properties, their surfaces are readily modified, and their production costs are lower than those of proteases.^[^ ^19^ ^]^ Hence, the noble metal nanoclusters and nanoparticles offer an elegant solution to meet the demand for simple and low‐cost diagnostic readouts due to their ability to function as catalysts to disproportionate or decompose hydrogen peroxide (H_2_O_2_).^[^ ^19^, ^20^ ^]^ Accordingly, in the present study, we sought to design ultra‐stable Au@Pt nanoflowers (nanofibers structure, NFs) with optimal catalytic activity for direct MetaSPR readout quantification of disease status in combination with a nanosensor platform. Nanozymes are not only used for catalytic amplification in sensors but also as enhanced labels for MetaSPR signals, replacing Au nanoparticles (AuNPs) for use in the process of signal amplification.^[^ ^21^ ^]^

In this study, we developed a nanozyme‐linked immunosorbent surface plasmon resonance (nano‐ELISPR) biosensor integrating the low‐cost nano cup sensor with Au@Pt NFs, for ultrasensitive, rapid, and portable testing of three cancer biomarkers serum levels, including AFP, carcinoembryonic antigen (CEA), and carbohydrate antigen 125 (CA125). The nano‐ELISPR platform combines the advantages of ELISA and MetaSPR technologies to facilitate the detection of analytes through homogeneous immunoassay etching reactions without requiring separation or amplification steps. Multilayer thin metal was evaporated on the substrate of the nanocup array to stimulate the MetaSPR signal. The oxidized 3,3',5,5'‐Tetramethylbenzidine (oxTMB) was formed by the nanozyme bound to the sensor. Simultaneously, an etching reaction occurs rapidly on the chip surface. The concentration of cancer biomarkers in serum can be accurately quantified by measuring the absorption spectrum changes of chip surface etching before and after the reaction with an optical device. In addition, the results indicate that the Au@Pt NFs have excellent peroxidase activity, potentially enabling them to replace the widely used horseradish peroxidase (HRP). Due to its robust stability, we predict that this method will also circumvent the cold transport challenges common to currently used immunochromatographic lateral flow assays.^[^ ^22^ ^]^ Hence, the developed nano‐ELISPR platform holds great promise for the early detection of cancer biomarkers in clinical serum samples.

## 2 Results and Discussion

### 2.1 Theory of Nano‐ELISPR Platform Detection of Biomarkers

The need for rapid, accurate, and convenient detection of cancer biomarkers presents significant challenges in monitoring and prognosticating disease. In countries with limited resources and large populations, the costs of ultrasensitive biomarkers are prohibitive.^[^ ^23^ ^]^ However, current ultrasensitive detection strategies often require complex instrumentation that may not be available in laboratories with relatively few resources.^[^ ^24^ ^]^

The MetaSPR chip was composed of titanium (Ti), gold (Au), and silver (Ag), while Ag and Au were ideal substrates for nanoarray plasma enhancement. Ti is used as the adhesive layer between polyethylene terephthalate (PET) and metal, and the double‐layer structure of Au and Ag is used as the excitation layer of the sensor signal of MetaSPR. The composite MetaSPR sensor prepared by laser interferometric lithography boasts reliable, cost‐effective, and convenient high throughput.

MetaSPR is a novel qualitative and quantitative detection technology that differs entirely from SPR and LSPR. That is, MetaSPR primarily uses the resonance coupling between incident light and metal nanostructures and utilizes the sensitivity of the SPR wavelength to the dielectric environment around the nanostructures to detect biochemical reactions. MetaSPR has an extraordinary optical transmission (EOT) effect, which refers to the appearance of light when it passes through a metal film with an aperture smaller than the wavelength of light.^[^ ^25^ ^]^ When the thickness of the noble metal coating differs, the absorption spectrum peak also differs. Hence, the nano‐ELISPR platform alters the noble metal thickness through the etching reaction of target molecules adsorbed on the substrate surface, to change the absorption spectrum peak. As such, the nano‐ELISPR serves as a portable, high throughput biosensor that does not require complex optical systems, like conventional SPR technology and has higher sensitivity than LSPR technology.

**Figure 1:**
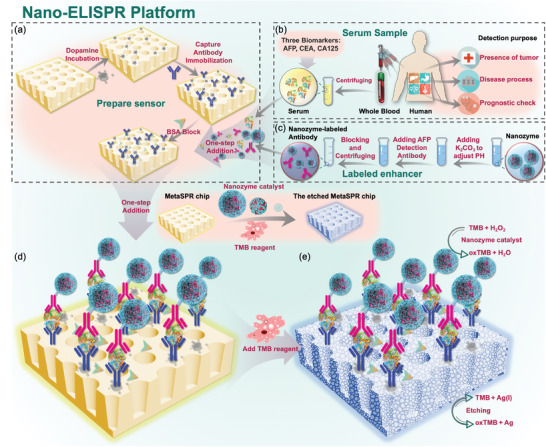
Schematic of rapid and ultrasensitive nano‐ELISPR platform used for detecting biomarker indicators. a) Modification and functionalization of MetaSPR chip surface. b) Serum samples were extracted to detect three biomarkers. c) Nanozyme‐labeled markers to D–Abs. d) One‐step addition of biomarkers and enhancers in serum. e) Quantitative detection of biomarkers by the etching reaction.

In the present study, oxTMB is used to etch the metal on the surface of the nano‐ELISPR biochip, causing the weakening, or disappearance, of the spectral peaks. To observe changes in the spectral peak and improve the detection sensitivity, a thin metal layer was plated on the nano‐ELISPR biochip. That is, the chip surface was functionalized to efficiently incubate antibodies and specifically capture antigens (**Figure** **1**a). Antigen and enzyme‐labeled antibodies (Figure 1b,c) were simultaneously added to the sensor well and the reactants reacted rapidly (Figure 1d). During the detection process, 3,3',5,5‐Tetramethylbenzidine (TMB) was first reacted under the catalysis of the enzyme to generate oxTMB, forming a blue peak. During the reaction progress, the blue peak disappeared following the etching reaction between the oxTMB and the chip. Meanwhile, the relative optical density (OD) value—that is, the absorption spectral peak of the chip—also significantly decreased (Figure 1e). The higher the concentration of oxTMB catalyzed by the enzyme (nanozyme or HRP) on the detection antibody (D‐Ab), the more obvious the etching reaction with the precious metal on the chip surface, and the lower the absorption peak of the chip.

### 2.2 Construction of the Nano‐ELISPR Biosensor

**Figure 2:**
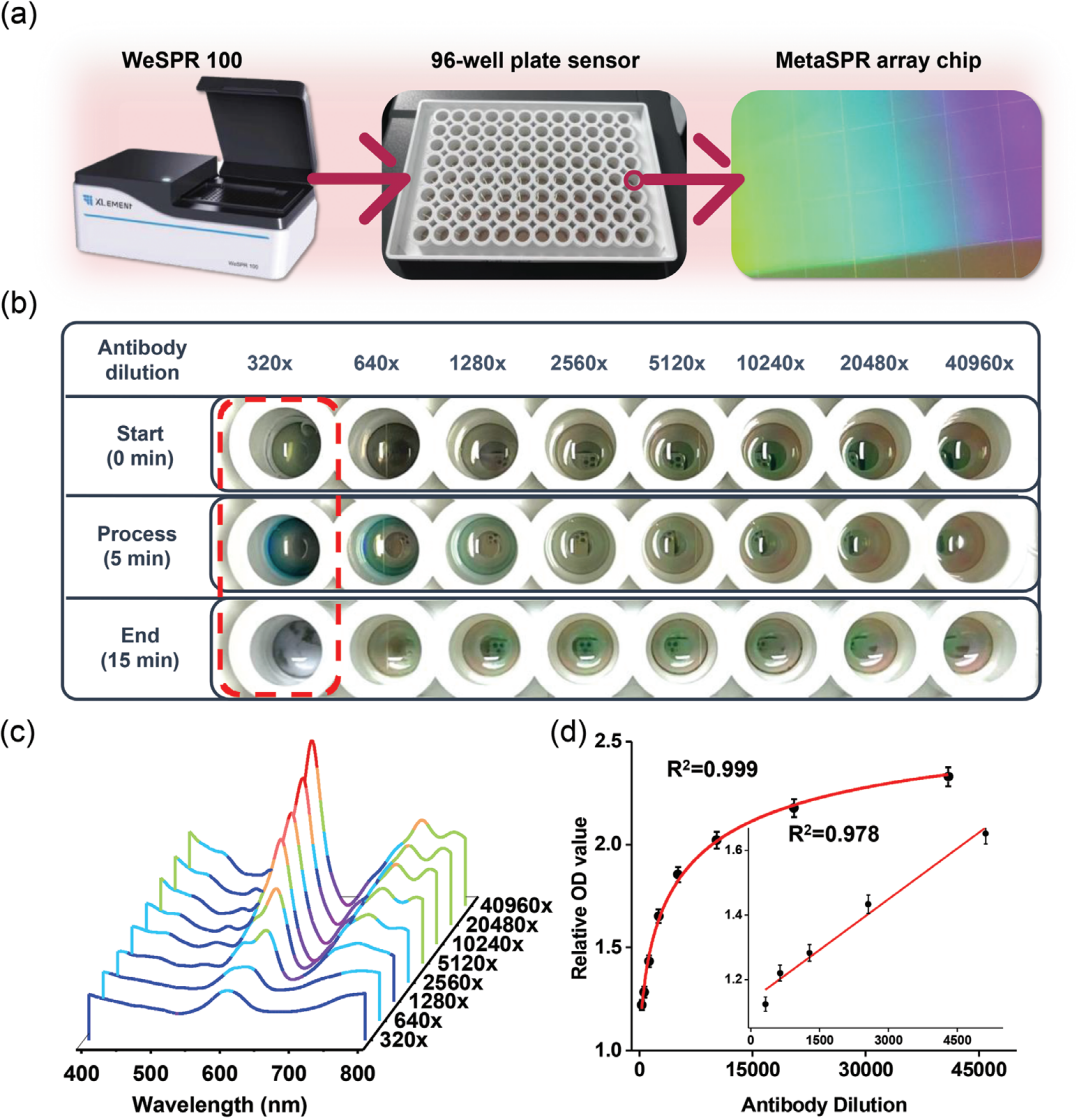
Nano‐ELISPR biosensor detection platform and verification of the etching reaction between oxTMB and the sensor. a) The multifunctional molecular analyzer used to measure changes in OD value (left); 96‐well plate reaction platform of nano‐ELISPR biosensor (middle); chip in each well emits colored light waves visible to the naked eye under full light illumination (right). b) Different HRP concentrations incubated on the sensor to demonstrate changes in the etching reaction of the sensor. c) Relative full spectrum of nano‐ELISPR biosensors catalyzed by different concentrations of HRP. d) Standard curve of peak shift value at 620 nm. Each value represents the mean of three independent experiments (*n* = 3).

The nano‐ELISPR biosensor is a high‐throughput 96‐well plate (**Figure** **2**a: middle panel) combined with a multifunctional molecular analyzer (XLement WeSPR 100) used to measure OD value changes (Figure 2a; left panel). The optical chip (Figure 2a; right panel), prepared by the replication molding process, was integrated with a 96‐well plate, from which the bottom was removed, to constitute the nano‐ELISPR biosensor.

To verify the etching effect of the MetaSPR chip, we diluted the HRP‐labeled AFP antibodies from the original concentration of 1 mg mL^−1^ from 320 to 40960 times, added the different dilutions to the different chip microwells, and incubated for 20 min (chip parameters: 9 nm Ti + 40 nm Ag + 10 nm Au). After the TMB reagent was added once, three images were captured at specific time intervals (0, 5, and 15 min) to record the color changes of chip holes at different concentrations (Figure 2b). After discarding the different concentrations of HRP‐labeled antibody solution, washing twice, and adding TMB reagent for 5 min, the chip wells showed varying degrees of blue colors. As the reaction approached 15 min, the blue color faded and the chip surface was gradually etched. The chip surface with the highest HRP concentration had the highest degree of etching, which was visible to the naked eye (Figure S1, Supporting Information). That is, the lower the concentration of HRP labeled antibody, the weaker the ability to catalyze TMB to generate oxTMB, and the less obvious the etching effect on the chip surface.

In addition, the spectrum was measured from 400 to 800 nm, resulting in the formation of a full spectrum with a clear gradient in peak change (Figure 2c). The most obvious absorption peak was at 620 nm. The OD offset value was negatively correlated with the concentration of the antibody incubated on the chip. Following 40960‐fold dilution of the antibody, a spectrum that was consistent with the original full‐spectrum was observed after completion of the sensor reaction. However, significant etching was observed after the 320‐fold diluted antibody reacted on the sensor surface. That is, after the sensor reaction, the chip etching reaction was intense, and the obtained absorption peak was low. Hence, the MetaSPR effect was weakened and the signal disappeared, resulting in a clear peak before and after the reaction. A four‐parameter logistic (4 PL) fit curve was constructed using the OD offset at 620 nm, resulting in a standard sigmoidal curve with a correlation coefficient (R^2^) of 0.999 (Figure 2d).

The experiment was repeated using 9 nm Ti + 50 nm Au chips. The sensor chip surface was cleaned several times after the reaction was completed and nearly identical spectral curves were obtained from the different chip wells coated with different antibodies (Figure S2, Supporting Information). The results indicate that a layer of Ag is required on the chip to enhance the etching of oxTMB on the chip surface.

### 2.3 Enhanced Performance of the Nano‐ELISPR Platform

To protect the metal layer on the surface of the sensor from being damaged, it was necessary to select an ideal primer to be deposited spontaneously on the substrate. Mussel‐inspired polydopamine (PDA) coatings are used extensively for membrane surface engineering due to their facile and versatile properties.^[^ ^26^ ^]^ PDA can be formed on virtually any substrate surface, including metals, metal oxides, ceramics, synthetic polymers, and other hydrophilic and hydrophobic materials.^[^ ^27^ ^]^ Under mildly alkaline conditions, where PDA was used as a primer layer, the substrate was immersed in a dilute aqueous solution of PDA buffered to an alkaline pH, resulting in the spontaneous deposition of PDA films on the substrate.^[^ ^28^ ^]^ The surface morphology of the chip changed from smooth to rough,^[^ ^29^ ^]^ and the PDA layer provided sufficient hydroxyl groups for subsequent experiments,^[^ ^30^ ^]^ thus, improving the modification efficiency of antibodies immobilized on the chip surface and enhancing sensor sensitivity. Figure S3, Supporting Information shows that the surface of the chip was modified with three concentrations of dopamine (0.2, 0.4, and 0.8 mg mL^−1^) to detect different concentrations of AFP (0–1.6 ng mL^−1^). When the surface of the chip was modified with dopamine, the reaction signal was proportional to the AFP concentration value. It is observed from Figure S3, Supporting Information that when the chip surface is modified with 0.4 mg mL^−1^ dopamine, a higher Meta SPR response value can be obtained both in high and low AFP detection concentration groups. Therefore, the chip surface was coated with 0.4 mg mL^−1^ dopamine. The coated chip and a bare chip without modification and with antibodies, were reacted with different concentrations of AFP. The modified dopamine chip with 0.4 mg mL^−1^ dopamine proved to be a favorable detection platform (Figure S4, Supporting Information).

**Figure 3:**
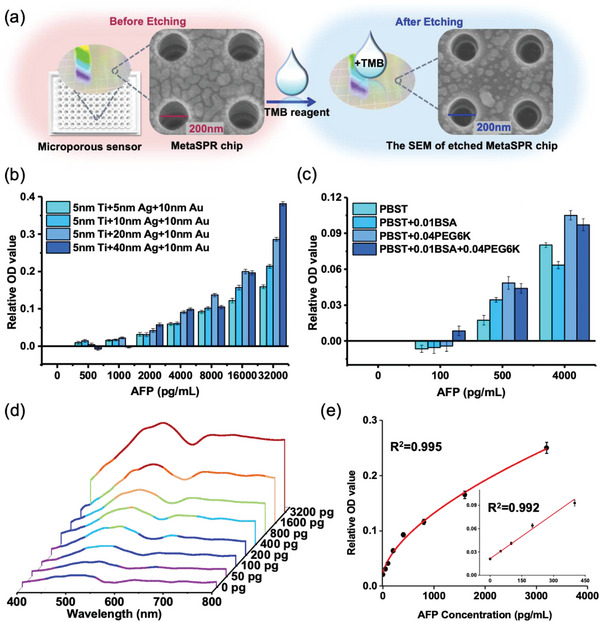
Optimization of the nano‐ELISPR biosensor. a) Image of a small aperture sensor plate (left); SEM image of the chip showing the replicated nano cup array (middle); SEM image of etching chip surface reaction with the nano–ELISPR sensor (right). b) Optimization of the 3D chip thickness on the nano‐ELISPR biosensor. c) Optimization of the sample reaction diluent on the nano‐ELISPR biosensor. d) Detection of the full AFP spectrum (concentration range of 50–3200 pg mL^−1^) by the optimized nano‐ELISPR biosensor. e) Standard curve of peak offset value at 620 nm. Each value represents the average of three independent experiments (*n* = 3).

To improve the detection sensitivity of cancer biomarkers, the analyte must react with the captured antibody (C‐Ab) on the chip in a small and precise region. Therefore, we used a 3D printer to design micro‐96‐well chip plates with a diameter of 2, 4, or 4 mm special sector. The samples were added to the bottom of the detection well for comparative experiments (Figure S5, Supporting Information). Figure S6, Supporting Information shows that the sensor with a smaller aperture (2 mm) improved detection sensitivity and was, therefore, applied to the design of the pocket bottomless 96‐well chip plate for the optimal nano‐ELISPR platform (**Figure** **3**a). That way, the platform reduces the cost associated with coating antibodies while improving the detection of analytes in small samples.

The surface of the sensor chip showed a well‐reproducible nano cup structure with a diameter of 200 nm under a scanning electron microscope (SEM); the smooth nanostructures are regular 3D cup‐shaped and uniform in size (Figure S7, Supporting Information). Before and after adding the TMB solution, the metal coverage morphology of the chip surface is obviously different (Figure 3a; left and right panels). The metal after the chip reacts with oxTMB is obviously less than that of the original chip, which results in the change of the MetaSPR signal.

The reaction principle of the nano‐ELISPR biosensor is the oxidation–reduction reaction between the Ag layer and oxTMB. Hence, chip thickness is a key factor influencing detection sensitivity. Therefore, we designed three film sensors with different thicknesses, which altered the thickness of the Ag layer parameter (including 40, 30, 20, and 10 nm) without impacting the thicknesses of the 9 nm Ti layer parameter and 10 nm Au layer parameter. Based on the nano‐ELISPR platform, we believe that the strength of the MetaSPR signal is closely related to the metal thickness of the chip, mainly because the thicker the chip, the more signals can be excited. However, the thicker the chip, the less sensitive it will be to the slight etching of the chip surface. When detecting 32 ng mL^−1^ AFP, the thicker chip exhibited the strongest EOT effect and highest OD value (Figure 3b), that is, the 5 nm Ti + 40 nm Ag + 10 nm Au chip. However, oxTMB influenced smaller etch reactions in the chip with increased chip thickness. That is, a thicker chip reduced the detection sensitivity when the limit of detection (LOD) value was 2000 pg mL^−1^. Meanwhile, the other three chips detected concentrations as low as 500 pg mL^−1^. More specifically, the 5 nm Ti + 10 nm Ag + 10 nm Au sensor exhibited the highest change in absorbance value and the widest detection range, which is conducive to the optimization of subsequent biosensors.

Similarly, selecting an optimal sample diluent is essential for improving sensitivity and preventing nonspecific reactions. Adding protein to the diluent is a common practice, for example, bovine serum albumin (BSA) is a stabilizer used to inhibit false positives. PEG6K is a polymerization accelerator that increases the reaction speed and binding efficiency of analytes to the chip plate.^[^ ^31^ ^]^ For the three sample diluents, BSA and PEG6K reagents were added to phosphate‐buffer saline (PBS) with 0.1% Tween 20 (PBST) to distinguish three concentration gradients and the negative control (Figure 3c). PEG6K improved the detection sensitivity of the sensor, while BSA inhibited the immune binding reaction and reduced nonspecific reactions. Hence, we selected the PBST buffer with 0.01% BSA and 0.04% PEG6K as the sample diluent.

After fixing the AFP C‐Ab on the biosensor surface, different concentrations of AFP samples (50–3200 pg mL^−1^, 25 µL per well) and D‐Ab (25 µL per well) were added to react for 20 min. The wells were then washed thrice, and 50 µL of the TMB and H_2_O_2_ mixture was added to verify the quantitative detection ability of AFP (≤ 40 min total). The dilution solution without AFP antigen was used as a negative control. After the etching reaction, the absorption spectra of different AFP sample concentrations exhibited a gradient decrease. Particularly, the resonance wavelength at 620 nm weakened the MetaSPR signal of the array chip (Figure 3d). The spectral curve of the control group exhibited less change without the etching reaction occurring. The standard curve of the relative OD value for AFP concentrations (50–3200 pg mL^−1^ range) is shown in Figure 3e. The standard curve was obtained using a 4 PL regression model, and the R^2^ was 0.995. Low concentrations (50–400 pg mL^−1^ range) also used the linear model to obtain an R^2^ of 0.992. Currently, the detection limit of the nano‐ELISPR biosensor platform for AFP detection is estimated to be 50 pg mL^−1^.

### 2.4 Characterization and Working Principles of the Nanozymes in Combination with the Nano‐ELISPR Biosensor

**Figure 4:**
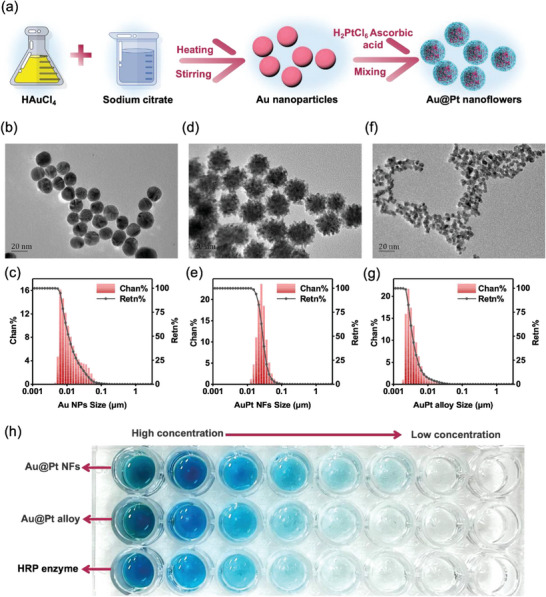
Characterization of Au@Pt NFs using nano‐ELISPR biosensors. a) Preparation of Au@Pt NFs. b) TEM image of AuNPs. c) Histogram of the particle size distribution of AuNPs. d) TEM image of Au@Pt NFs. e) Histogram of the particle size distribution of Au@Pt NFs. f) TEM image of Au@Pt alloy. g) Histogram of the particle size distribution of Au@Pt alloy. h) Catalytic color reaction of three enzymes. (Chan% represents the proportion of the number of particles in a certain size range to the total, and Retn% represents the sum of 100% minus the cumulative proportion.)

To further improve the detection sensitivity of the nano‐ELISPR sensor, we developed several artificial nanozymes with improved catalytic stability and higher catalytic activity to replace HRP. Au@Pt NFs were prepared using a seed‐mediated growth method.^[^ ^32^ ^]^ In preparing the Au@Pt NFs, AuNPs were first prepared by rapidly adding sodium citrate to the HAuCl_4_ solution, followed by stirring and heating, forming a golden‐red solution (**Figure** **4**a). Ascorbic acid was subsequently added to the boiling AuNP solution, followed by H_2_PtCl_6_, and finally, the Au@Pt NFs were obtained (Figure 4a and Figure S8, Supporting Information). Au seeds (AuNPs) exhibited a smooth surface and spherical shape under transmission electron microscopy (TEM; Figure 4b), and the calculated average AuNPs size was 10.87 nm, as indicated by the Nano Measure software. The diameter of AuNPs measured by Microtrac's three‐laser particle size analyzer was 11.09 nm. As shown in Figure 4c, the Chan% histogram is mainly concentrated between 6.39 and 15.19 nm, and the Retn% curve shows a particle size distribution of 5.37–17.19 nm. This demonstrates the homogeneity of AuNPs suitable for use in subsequent experiments. Through TEM morphology and particle sizer characterization, after growing Pt nanowires on the basis of AuNPs, the synthesized Au@Pt NFs were observed to form flower shapes under TEM (Figure 4d). The sizes calculated by the software and particle size analyzer were 23.67 and 28.11 nm, respectively. As shown in Figure 4e, Chan% indicates that the particles are concentrated from 18.06 to 36.1 nm, while Retn% indicates that the particles are distributed from 15.19–102.2 nm. In addition, Au@Pt alloy is also commonly used as a nanozyme. Figure 4f shows the uniformity of Au@Pt alloys particles under TEM and a particle size of 3.02 nm by software fitting. The diameter of Au@Pt alloy measured by the particle size analyzer was about 3.55 nm, and the results were consistent. Chan% and Reta% parameter analysis revealed Au@Pt alloy particles concentrated in the range of 2.26–3.8 nm, with an overall distribution of 1.9–43 nm (Figure 4g). The nanozyme material has good morphology and uniformity and can be used in the nano‐ELISPR platform.

To verify the effect of the two nanozymes (Au@Pt NFs, Au@Pt alloys) and HRP, we unified the antibody concentration of the labeled enzyme and added the chromogenic reagent to the transparent microtiter plate. The two nanozymes all exhibited catalytic activity superior to that of HRP (Figure 4h). Due to the good stability of nanozymes, the three enzymes were placed at different temperatures for the enzyme activity test. Figure S10, Supporting Information shows that the nanozyme activity is higher than that of the HRP enzyme at high temperatures. Due to the low Au@Pt alloy volume, the electrostatic adsorption generated during antibody labeling is weak, making it more difficult to label antibodies, which would affect the high sensitivity detection of the nano‐ELISPR platform; therefore, we used Au@Pt NFs as the preferred nanozyme in the follow‐up experiments.

### 2.5 Quantitative Determination of the Nano‐ELISPR Biosensor

**Figure 5:**
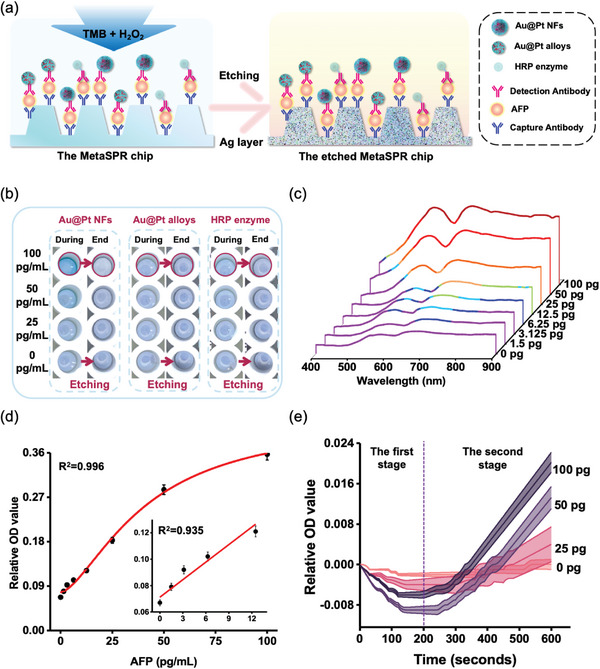
Optimization and verification of the nano‐ELISPR biosensor detection platform. a) Scheme of enzyme catalytic reaction of TMB and H_2_O_2_. b) Before and after images of the etch reaction for the three catalytic enzymes. c) Full AFP spectrum (400–900 nm) at different concentrations (0–100 pg mL^−1^). d) Standard curve for the detection of AFP using enhanced nano‐ELISPR (R^2^ = 0.996; LOD = 1.5 pg mL^−1^, about 21.74 fM) over 600 s. Each value represents the mean of three independent experiments (*n* = 3). e) Dynamic binding curves of AFP at different concentrations.

The surface Pt nanowires endow Au@Pt NFs or Au@Pt alloy with ultra‐high peroxidase‐like catalytic activity, which can be used as the catalytic reagents to enhance the detection sensitivity of the nano‐ELISPR platform (**Figure** **5**a). Highly sensitive detection performance was achieved by adding a chromogenic substrate to the sensor well, which was catalyzed by the nanozymes to generate oxTMB (Figure 5b). The etching reaction of the chip surface before and after the catalytic reaction of the three enzymes can be observed. The change in the chip with the high concentration of 100 pg mL^−1^ is highlighted by a circle, and a difference can be seen between the chip during the reaction (react for 5 min) and the chip surface with faded blue (end for 15 min). Based on the results, Au@Pt NFs were selected as the catalytic enzyme with the highest activity.

Rapid quantitative detection of AFP samples of a much lower concentration range (0–100 pg mL^−1^) was achieved via a single‐step Au@Pt NFs‐enhanced nano‐ELISPR sensor based on a micro‐96‐well chip plate device. The absorption spectra of the wells containing different AFP concentrations showed an obvious gradient (Figure 5c). In addition, the 4 PL curve fitting R^2^ between the relative change in OD values and the AFP concentration (0–100 pg mL^−1^) was 0.996 (Figure 5d). In the typical relative absorption spectra of the nano‐ELISPR sensor chip, signals appeared at 580 nm (Figure 5d). Additionally, dynamic curves for the different AFP concentrations (0, 25, 50, and 100 pg mL^−1^) and the control sample were apparent at 580 nm and 600 s (Figure 5e). In the first stage, within 120 s, the dynamic curves exhibited a downward trend due to the color of oxTMB. In the second stage (200 s later), different concentrations of AFP increased and exhibited significant differences (Figure 5e). The AFP concentration is related to the degree of the MetaSPR peak blue shift and the etching reaction. Therefore, the developed nano‐ELISPR biosensor realized rapid quantification of cancer markers with ultra‐high sensitivity under optimal conditions, suggesting that the sensor has great potential in the field of early cancer screening.

### 2.6 Performance Verification of the Nano‐ELISPR Biosensor

Biosensor specificity can be compromised when analytes are detected in complex matrices, such as human blood. Hence, the specificity of the nano‐ELISPR immunoassay was investigated by detecting potential interferences and common biomarkers, including prostate‐specific antigen (PSA), CEA, chorionic gonadotropin (HCG), C‐reactive protein (CRP), hepatitis B surface antigen (HBsAg), and hepatitis Be antigen (HBeAg) (Figure S11, Supporting Information). When the AFP sample was added, the relative OD value increased significantly, whereas, following the addition of the interferent, the signal was relatively similar to that of the blank control. No false positive results were observed in the presence of different interferences, highlighting the high specificity of the proposed nano‐ELISPR immunoassay for AFP.

The long‐term stability of the assay was further investigated under different storage times and temperature conditions. The detection results of the proposed nano‐ELISPR sensor were consistent with the initial response after 3 and 7 days of storage, implying that the developed immunosensor could maintain sufficient stability for AFP detection (Figure S12, Supporting Information). Furthermore, the accelerated test results were investigated at different temperatures, which demonstrated the storage stability of the nano‐ELISPR sensor for AFP detection (Figure S13, Supporting Information). The nano‐ELISPR biosensor showed good repeatability between different chip plates, or within the same plate. Hence, the nano‐ELISPR biosensor assay may have additional applications in clinical sample detection while also improving detection efficiency and simplifying the operational process.

### 2.7 Comparison of Nano‐ELISPR and ELISA Capabilities for Detecting AFP

**Figure 6:**
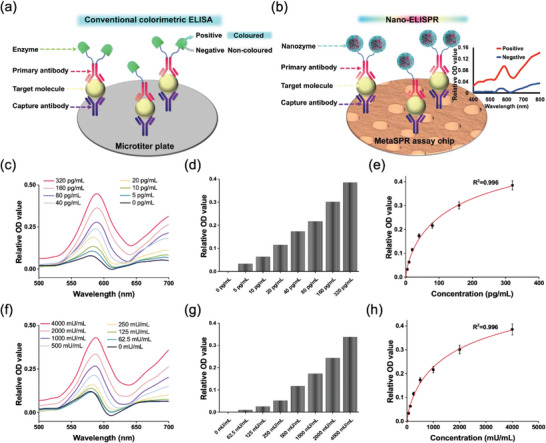
Verification of detection accuracy using the nano‐ELISPR sensor. a) ELISA detection platform process. b) Nano‐ELISPR biosensor detection platform process. c) Differential spectra of CEA at 500–700 nm at different concentrations (0–320 pg/mL). d) Relative OD values at 580 nm with different sample concentrations. e) Standard curve of CEA detection by the nano‐ELISPR platform (R^2^ = 0.996). Each value represents the mean of three independent experiments (*n* = 3). f) Differential spectra of CA125 at 500–700 nm at different concentrations (0–4000 mU/mL). g) Relative OD values at 580 nm with different sample concentrations. h) Standard curve of CA125 detection by the nano‐ELISPR platform (R^2^ = 0.996). Each value represents the mean of three independent experiments (*n* = 3).

**Figure** **6**a,b compare the entire reaction principal process of commercial ELISA and highly sensitive short‐time nano‐ELISPR biosensors. In a traditional colorimetric ELISA, the target molecule is captured by a specific antibody on a disposable substrate, followed by a sandwich reaction with an enzyme‐labeled D‐Ab (Figure 6a). The signal is generated via the conversion of an enzymatic substrate to a colored molecule. The intensity of the solution color is terminated with a stop solution and quantified by measuring absorbance with a plate reader. The reaction time is at least 3 h. Meanwhile, the nano‐ELISPR biosensor optimized the method by reducing the volume of the sample added (≈20 µL), increasing sensitivity, and shortening the reaction time to 40 min (Figure 6b).

Moreover, compared with one‐step quick‐ELISA, nano‐ELISPR reduced nonspecific binding caused by HRP instability and improved the sensitivity and stability of biomarker detection. Human serum samples were obtained from the hospital to further demonstrate the feasibility and practicality of the established strategy for testing real samples. A certain amount of standard substance was added to the same sample for determination. The measured value of the sample was then deduced from the measured result, and the recovery rate was calculated. Based on the standard addition method, a series of standard AFP antigens to 1000, 4000, and 16000 pg mL^−1^ (same concentration range used for ELISA), were added and analyzed (**Table** **1**). The recovery rate of the same sample was between 79.3% and 115.3% for all two methods, indicating that they all exhibit good stability and accuracy. The recoveries of the developed nano‐ELISPR platform were between 84.3% and 115.3%, indicating that the sensor may have high accuracy in detecting AFP in clinical samples.

**Table 1:** Recovery of the quick‐ELISA and nano‐ELISPR assay for AFP antigen detection

Concentration [pg mL^−1^]		Quick‐ELISA		Nano‐ELISPR	
		Average [pg mL^−1^]	Recovery [%]	Average [pg mL^−1^]	Recovery [%]
1000	Serum1	896.7	89.7	1152.5	115.3
	Serum2	792.5	79.3	1050.9	105.1
4000	Serum1	3276.5	81.9	4126.9	103.2
	Serum2	3595.6	89.9	3372.5	84.3
16 000	Serum1	12 604.2	78.8	15 924.3	99.5
	Serum2	12 771.6	79.8	14 069.9	87.9

### 2.8 Multifunction of Nano‐ELISPR Biosensor for Other Cancer Biomarkers

In order to verify the universality and demonstrate the applicability of the nano‐ELISPR platform for the detection of other biomarkers, we detected two different indicators CEA and carbohydrate antigen 125 (CA125). Normally, CEA is present at very low levels in the blood; however, an increase in its concentration indicates pathological changes or trauma of the prostate.^[^ ^33^ ^]^ We applied it to CEA C‐Ab modified sensor surface and Au@Pt NF labeled with CEA D‐Ab, which was used to detect CEA antigen. In the typical differential absorption spectrum of a nano‐ELISPR sensor chip, the signal appeared at 580 nm (Figure 6c), and the change in OD values, proportional to the different CEA concentrations, was observed at 580 nm at 15 min (Figure 6d). In addition, the relative OD value at 580 nm was obtained from the full spectrum with respect to the 4PL model of CEA with different concentrations, resulting in an R^2^ value of 0.996 in the 0–320 pg mL^−1^ range (Figure 6e).

Ovarian cancer is the second most lethal gynecological malignancy. Cancer marker CA125 has been used as the primary ovarian cancer marker to diagnose and screen for stage I and II ovarian cancers.^[^ ^34^ ^]^ CA125 is not only a non‐specific inflammatory marker but is also directly involved in cardiovascular diseases such as inflammation and atherosclerosis and is a powerful predictor and risk factor for cardiovascular diseases. CA125 protein biomarkers were detected with the nano‐ELISPR platform with similar methods used for AFP and CEA. Figure 6f shows the typical differential full absorption spectrum of the nano‐ELISPR sensor chip etch signal at 580 nm. The relative OD value at 580 nm decreased with CA125 concentrations (Figure 6g); the constructed 4 PL standard curve had an R^2^ value of 0.996 with a 0–4000 mU mL^−1^ CA125 range (Figure 6h).

### 2.9 Validating the Accuracy of the Nano‐ELISPR Biosensor for Detecting Clinical Samples

**Figure 7:**
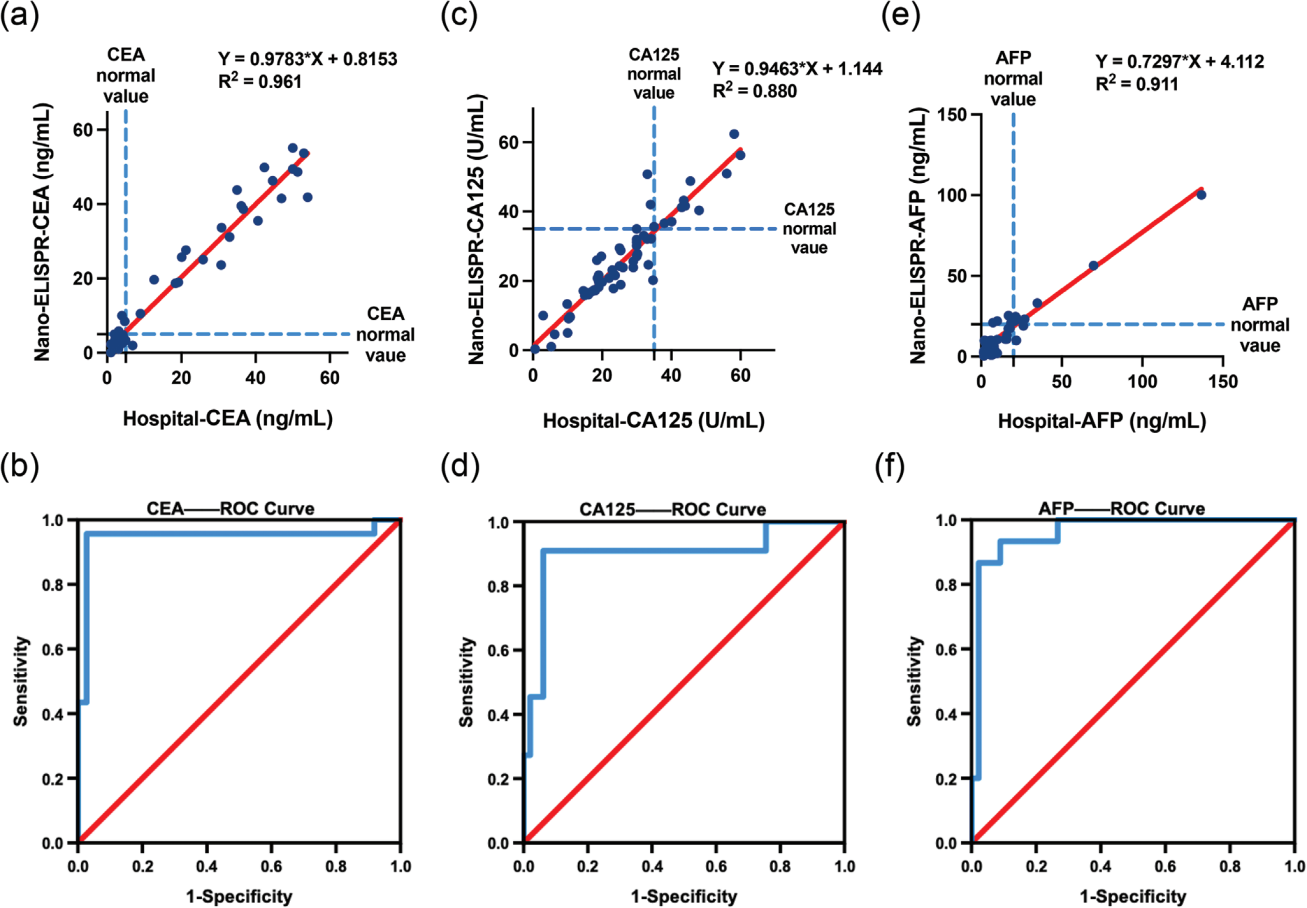
Clinical trial verification. a) Measurement and comparison of CEA biomarkers with different blood serum samples by nano‐ELISPR biosensor and the hospital results. b) ROC curve analysis of the nano‐ELISPR biosensor CEA results and the hospital results. c) Measurement and comparison of CA125 biomarkers with different blood serum samples by nano‐ELISPR biosensor and the hospital results. d) ROC curve analysis of the nano‐ELISPR biosensor CA125 results and the hospital results. e) Measurement and comparison of AFP biomarkers with different blood serum samples by nano‐ELISPR biosensor compared with the hospital results. f) ROC curve analysis of the nano‐ELISPR biosensor AFP results compared with the hospital results.

In order to verify the feasibility of the platform, we measured the CEA and CA125 biomarkers in real human serum samples, and the measured results were compared with the hospital test values. 60 clinical samples were selected from the data in **Figure** **7**a to test the CEA biomarker, and the results of the nano‐ELISPR biosensor chip test had a good correlation with the hospital report (R^2^ = 0.961, *Y* = 0.9783 × *X* + 0.8153). According to the corresponding ROC curve (Figure 7b), the critical diagnostic value is the hospital standard (5 ng mL^−1^), which can provide 95.7% sensitivity and 97.3% specificity for the detection of CEA biomarkers (**Table** **2**). In addition, as shown in Figure 7C, the CA125 biomarker also shows a good correlation (R^2^ = 0.880, *Y* = 0.9463 × *X* + 1.144). The clinical detection cutoff value is 35 U mL^−1^. From the ROC curve analysis, the nano‐ELISPR biosensor provides 90.9% sensitivity and 93.9% specificity for the detection of the CA125 biomarker (Table 2). In addition, as shown in Figure 7E, the AFP biomarker also shows a good correlation (R^2^ = 0.911, Y = 0.7297 × *X* + 4.112). The clinical diagnostic cut‐off value is 20 ng mL^−1^. From the ROC curve analysis, the nano‐ELISPR biosensor provides 86.7% sensitivity and 97.8% specificity for the detection of AFP biomarkers (Table 2).

**Table 2:** ROC curve analysis of nano–ELISPR to detect three biomarkers

Characteristic	Value of CEA	Value of CA125	Value of AFP
Sensitivity [%]	95.7	90.9	86.7
Specificity [%]	97.3	93.9	97.8
Area under the concentration–time curve	0.900	0.946	0.961
95% confidence interval	0.770–1.001	0.865–1.001	0.912–1.001

These results show that the nano‐ELISPR‐based immunoassay platform can be used for the very early detection of cancer protein biomarkers in clinical samples. Due to the high sensitivity of the method, clinical samples can be repeatedly diluted, thus reducing the interference of other substances. This method is simple and effective with a simple microplate reader, and has great potential in the application of biosensors in point–of–care diagnosis, especially for the rural population in countries or regions with scarce resources.

## 3 Conclusion

In summary, we proposed a fast, sensitive, and portable nano‐ELISPR biosensor based on the etching reaction process of nanozymes and oxTMB for the early detection of cancer biomarkers in serum. Compared with traditional plasma technologies including SPR or previously reported MetaSPR chips that require at least 70—90 nm Au layers, the nano‐ELISPR biosensor requires only 5 nm Au layer preparation to protect the Ag layer, which reduced the associated cost by nearly 20‐fold. In addition, compared to natural enzymes, nanozymes offer higher catalytic stability, easier modification processes, and lower manufacturing costs. Collectively, the nano‐ELISPR platform uses Au@Pt NFs‐labeled D‐Ab for intercalation detection, while the etching reaction acts as an amplification reaction, and the concentration of analytes is achieved in one–step mainly by the degree of MetaSPR signal attenuation after etching generated by oxTMB. Importantly, the anti‐AFP mAb‐functionalized nano‐ELISPR achieved ultra‐high sensitivity (21.74 fM) for the AFP molecule, several orders of magnitude higher than most label‐free assays, while successfully and specifically detecting AFP in human serum, which contains other interferers. Also, to verify the platform universality of nano‐ELISPR, CEA, CA125, and AFP biomarkers were detected in 60 clinical serum samples, which were in agreement with the hospital detection values. Therefore, the minute‐scale, high‐sensitivity, superior‐specificity nano‐ELISPR biosensor can be used as a potential method for early detection in monitoring physical health status, particularly those associated with clinical diagnostic applications and point‐of‐care diagnosis not only for cancer biomarkers but also for other chemical and biological processes.

## 4 Experimental Section

### Materials

BSA, PBS, and carbonate‐buffered saline (CBS) were purchased from Sigma‐Aldrich (St. Louis, MO, USA). H_2_O_2_ and TMB were obtained from Aladdin Biological Technology Co. Ltd. (Xi'an, China, www.aladdin‐e.com). Tween20 and dopamine hydrochloride were obtained from Shanghai Acro Organics (Shanghai, China, www.acros.com). Human AFP Matched ELISA Antibody Pair Set (catalog no. 12177) was purchased from Sino Biological Co., Ltd. (Beijing, China). Anti‐AFP mAb_1_ and anti‐AFP mAb_2_ were purchased from Medix Biochemica (Kauniainen, Finland). AFP, CEA, PSA, CA125, CRP, HBsAg, and HBeAg antigens were obtained from Fitzgerald (Acton, MA, USA). WeSPR 100 multifunctional analyzer and Quick‐Elisa Cancer Marker Multiplex kits are purchased from the XLement (Shanghai, China). All chemicals were used without further purification. Clinical blood samples were collected from the Union Hospital, Tongji Medical College, Huazhong University of Science and Technology (Wuhan, China). This study was approved by the Ethics Committee of the Science and Technology Department of Huazhong University of Science and Technology (certificate no. S1029).

### Fabrication and Characterization of 3D Nano Cup Array Chip

Nano cup arrays with a radius of 200 nm, height of 500 nm, and periodic arrangement of 400 nm were fabricated on a 12‐inch silicon wafer mold by laser interference lithography. The UV‐curable polymer solution was spread evenly on the clean silicon wafer nano cup array mold with a glue dispenser. PET was slowly attached to the mold surface and evenly coated with the glue sheets, ensuring that no bubbles formed, perfectly fitting between the two, and cured under UV light. The PET sheet was exfoliated from the mold along with the UV‐cured polymer with nano cup arrays to obtain the substrate. On the surface of the nano cup array PET, 5 nm Ti, 10–50 nm Ag, and 2–10 nm Au film were deposited using electron beam evaporation to form MetaSPR biochips, which were cut to the corresponding size and glued onto open‐bottom 96‐well plates created with a 3D printer (Objet30 Prime; Stratasys Ltd., Rehovot, Israel) to form the nano‐ELISPR biosensor.

### Surface Functionalization

First, the surface of the nano cup array chip was functionalized with a PDA film to immobilize the AFP C‐Ab. PDA was a mussel‐inspired adhesive, and antibodies could be firmly attached to the surface of the chip through a Schiff base reaction between the amino residues of the antibody and the quinone groups formed in PDA.^[^ ^35^ ^]^ For PDA coating, dopamine hydrochloride solution was diluted to 0.4 mg mL^−1^ with tris (10 mM, pH 8.5) solution; 20 µL was added to each well of the 96‐well plate chip and incubated for 10 min. The chip wells were rinsed twice with MilliQ water and dried with nitrogen; 50 µL of 8.0 µg mL^−1^ AFP C‐Ab in CBS (1×) was added to the chip wells and incubated for 4 h at 37 °C. The remaining unconjugated antibodies were then removed by rinsing with PBST and blocked with 50 µL of 1% BSA in PBS for 30 min at 37 °C. Finally, microarray plates with their surfaces incubated with coated antibodies were stored at 4 °C until further use.

### Preparation of Au@Pt NFs, Au@Pt Alloys, and Fabrication of Au@Pt NFs D‐Ab Conjugates

To synthesize Au@Pt alloys, 2.4 mL of 2.9 × 10^−2^ M HAuCl_4_ solution and 2.9 mL of 2.4 × 10^−2^ M K_2_PtCl_4_ solution were diluted in 92.2 mL of deionized water and stirred thoroughly. Subsequently, 1.5 mL of 3.9 × 10^−2^ M tri‐sodium citrate solution was added to the mixture and stirred vigorously; 1 mL of freshly prepared NaBH_4_ solution (4.0 × 10^−2^ M) was then added. The reaction solution was stirred for 12 h at 25 °C.

Au@Pt NFs were prepared using the seed‐mediated method. First, AuNPs were prepared, which were synthesized by citrate reduction of HAuCl_4_.^[^ ^36^ ^]^ The prepared AuNP solution was taken as seeds to grow Pt nanowires on the AuNPs surface.^[^ ^32a^ ^]^ Subsequently, 1 mL of ascorbic acid (0.1 M) was added to the seed solution and combined with 1.5 mL of H_2_PtCl_6_ (0.01 g mL^−1^). The solution was then placed at 25 °C for 1 h to generate Au@Pt NFs.

The AFP D‐Ab was labeled with Au@Pt NFs using a method similar to that previously described for labeling AuNPs with proteins.^[^ ^37^ ^]^ Briefly, 1 mL of Au@Pt NFs at a concentration of 0.48 mg mL^−1^ was added to PBS, and the pH value of the solution was adjusted to 7.8 and mixed well with ultrasound. Next, 5 µL of AFP D‐Ab (1 mg mL^−1^) was added to the solution and incubated for 30 min at 25 °C; 15 µL of blocking solution containing BSA (5%, w/v) was then added and incubated for 30 min. The solution was centrifuged at 5535× g for 20 min and the Au@Pt NF pellets were resuspended in 1000 µL of stabilization buffer (20 mM Tris [pH 9.1], 0.3% sucrose, and 0.05% PEG 20000). The conjugate was stored at 4 °C for subsequent experiments.

### Measurement Process

PBST buffer (150 µL) was added to the C‐Ab modified chip wells and washed twice. Then, 50 µL of PBST buffer was added to each well and placed in a universal microplate multifunctional analyzer (WeSPR 100, XLement) to record the initial (400–800 nm) OD value. Specific OD values at the reporting wavelength were recorded using the analyzer. Subsequently, 50 µL of the sample with various AFP concentrations were added to the biosensor (2 pg mL^−1^ or other sample concentration > 1.5 pg mL^−1^); the liquid was discarded after 15 min. Meanwhile, 50 µL of AFP D‐Ab (10 pg mL^−1^) was added to the wells of the sensor and allowed to react for 20 min; wells were then washed twice with 150 µL of PBST buffer each time.

Finally, 50 µL of a TMB solution and H_2_O_2_ solution mixture was added to the biosensor. The sensor was shaken at 700 rpm for 15 min and then placed in the analyzer to record the endpoint value. By subtracting the end OD wavelength (or specific band point) from the OD value of the start wavelength (or specific band point), a standard curve for quantifying AFP concentration after data analysis was created.

### Detection of AFP in Complex Sample Matrix using the Nano‐ELISPR Biosensor

By centrifuging the blood sample at 2500× g and 4 °C for 15 min, the precipitated plasma was removed, and only the clear serum was obtained for the subsequent experiment. Subsequently, various proteins, including AFP, were added to pre‐prepared healthy control serum samples used as clinical samples. Normal human serum without AFP antigen was used as an additional negative control. Before measurement, the OD value of the nano‐ELISPR biosensor chip immobilized with the specific C‐Ab was read. Subsequently, spiked serum samples and the enzyme‐labeled D‐Ab (volume ratio of 1:1) reacted in a 96‐well plate for 20 min. To verify the stability of the platform, the AFP recovery rate was calculated according to the following formula.

(1) $$\begin{array}{ccc} \mathrm{Recovery}\;\left(\%\right) & = & [(\mathrm{Concentration}\;\mathrm{detected}\;\mathrm{in}\;\mathrm{spiked}\;\mathrm{samples}\\ & & -\mathrm{Concentration}\;\mathrm{detected}\;\mathrm{in}\;\mathrm{unspiked}\;\mathrm{samples})/\\ & & \mathrm{spiked}\;\mathrm{concentration}\;\mathrm{in}\;\mathrm{samples}]\times 100\% \end{array}$$

### Analysis of Commercial ELISA

To demonstrate the functionality of the nano‐ELISPR platform, AFP concentrations were quantified using commercial ELISA kits and high‐sensitivity AuNP‐coupled MetaSPR sensors.^[^ ^38^ ^]^ First, AFP was quantitatively detected using a commercial ELISA kit (XLement Quick‐ELISA). Specifically, 100 µL of different concentrations of AFP antigen standards were added to a 96‐well plate immobilized with AFP C‐Ab. Afterward, 100 µL of diluted enzyme‐linked AFP D‐Ab solution was added to each well and reacted for 60 min with shaking (37 °C and 200 rpm). The wells were washed three times with the provided wash buffer (150 µL per well), and then 50 µL of substrate solutions A and B were added, and the reaction continued for 20 min in the dark. The reaction was stopped by adding 50 µL of 0.2 M H_2_SO_4_ to each well, and the OD at 450 nm (OD_450_) was recorded over 30 min using a microplate reader.

## Conflict of Interest

The authors declare no conflict of interest.

## Supporting information

Supporting Information

## Data Availability

The data that support the findings of this study are available in the supplementary material of this article.
