# Supplementary material for: Nanozyme‐Catalyzed Metasurface Plasmon Sensor‐Based Portable Ultrasensitive Optical Quantification Platform for Cancer Biomarker Screening
Source: Adv Sci (Weinh). 2023 Jun 26;10(24):2301658. doi: 10.1002/advs.202301658 (PMC10460869; doi:10.1002/advs.202301658)
Supplement: Supplementary file 1 — Supporting Information [file ADVS-10-2301658-s001.pdf]

ADVANCED SCIENCE
Open Access

Supporting Information

for *Adv. Sci.*, DOI 10.1002/advs.202301658

Nanozyme-Catalyzed Metasurface Plasmon Sensor-Based Portable Ultrasensitive Optical Quantification Platform for Cancer Biomarker Screening

Rui Li, Hongli Fan, Hanlin Zhou, Youqian Chen, Qingcai Yu, Wenjun Hu, Gang L. Liu and Liping Huang**

Nanozyme-Catalyzed Metasurface Plasmon Sensor-Based Portable Ultrasensitive Optical Quantification Platform for Cancer Biomarker Screening

Rui Li[1], *Hongli Fan*[1], *Hanlin Zhou*[2], *Youqian Chen*[1], *Qingcai Yu*[3], *Wenjun Hu*[1], *Gang L. Liu*[1,*], *Liping Huang*[1,2,*]

[1] College of Life Science and Technology, Huazhong University of Science and Technology, 1037 Luo Yu Road, Wuhan, 430074, P. R. China

[2] Biosensor R&D Department, Liangzhun (Wuhan) Life Technology Co., Ltd., 666 Gaoxin Avenue, Wuhan, 430070, P. R. China

[3] School of Life and Health Science, Anhui Science and Technology University, Fengyang, 233100, P. R. China

*Corresponding author, E-mail address: Gang L. Liu (loganliu@hust.edu.cn); Liping Huang (huangliping@liangzhun.com.cn)

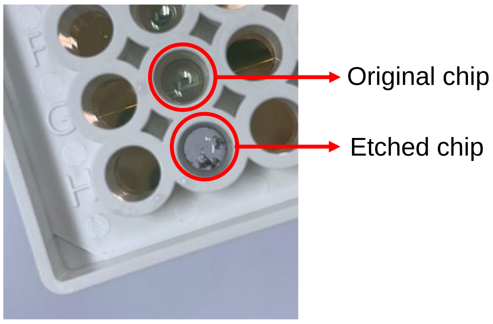

Figure S1. The etching reaction with the highest concentration HPR is observed by the naked eye.

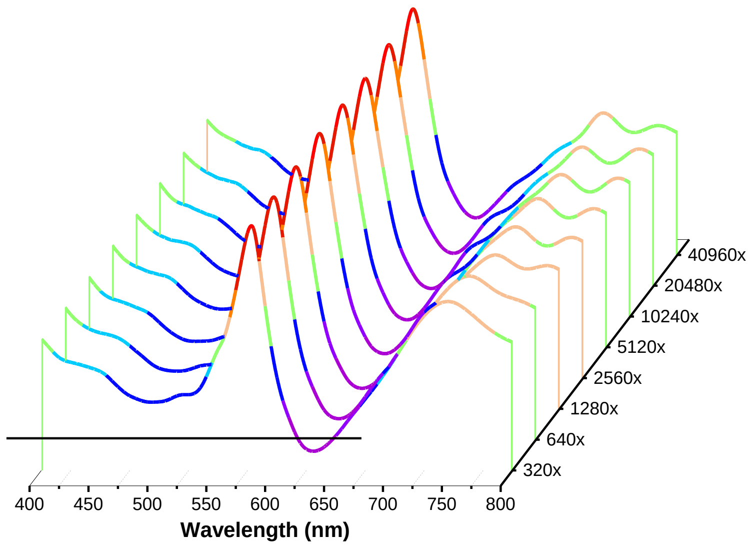

Figure S2. Relative full spectrum of Nano-ELISPR biosensors (9 nm Ti + 50 nm Au) catalyzed by different concentrations of HRP.

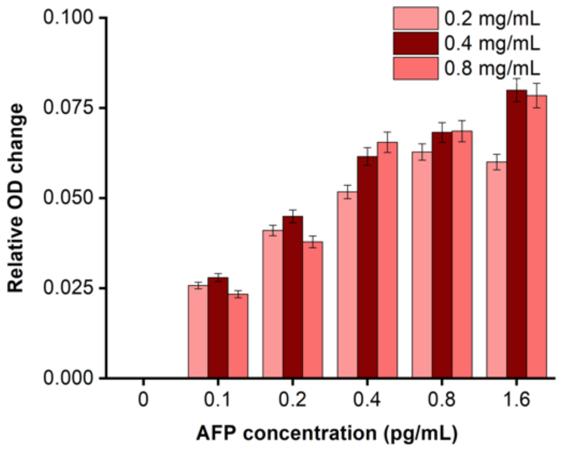

Figure S3. The surface of the chip is modified with three different concentrations of dopamine (0.2, 0.4, 0.8 mg/mL) to test AFP detection at different concentrations (0-1.6 pg/mL).

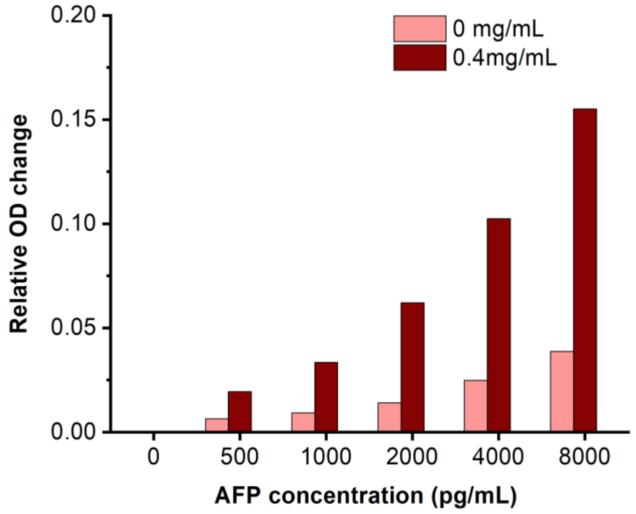

Figure S4. The optimal chip modification condition was reached.

a b c

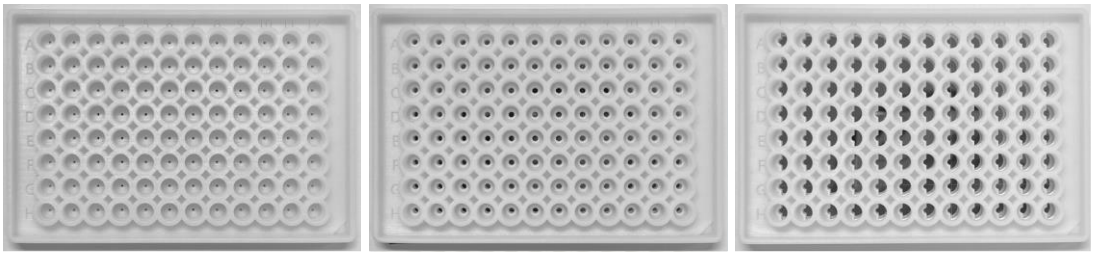

Figure S5. Different chip detection platforms with different shapes and diameters of holes.

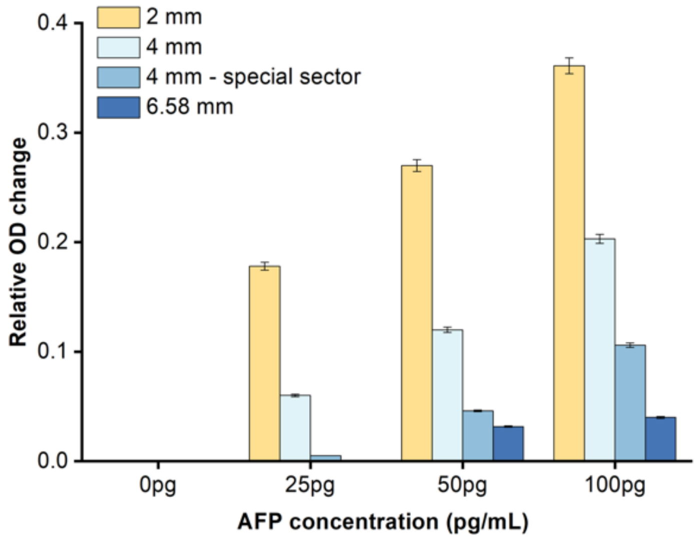

Figure S6. The different shapes of biosensor detection different concentrations of AFP.

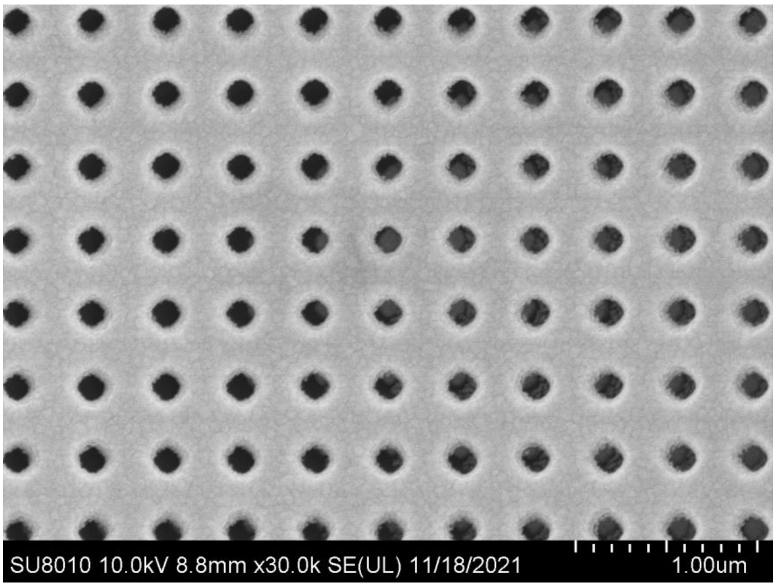

Figure S7. Uniform nano cup array under SEM.

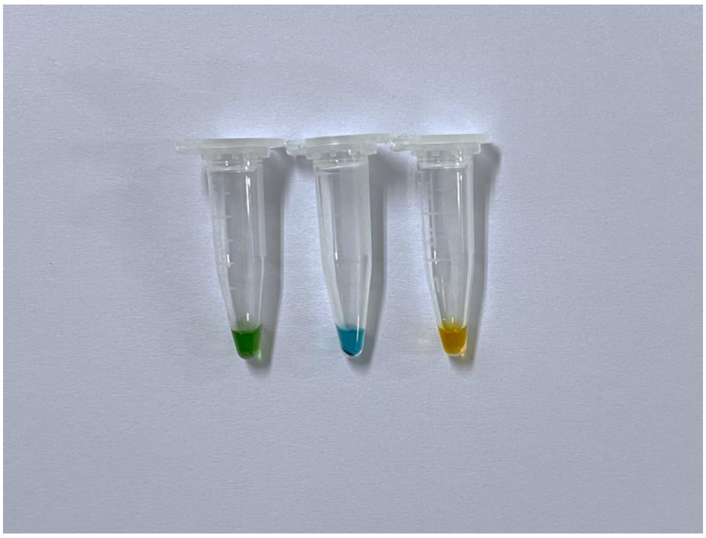

Figure S8. Color pictures of nanozymes with different concentrations.

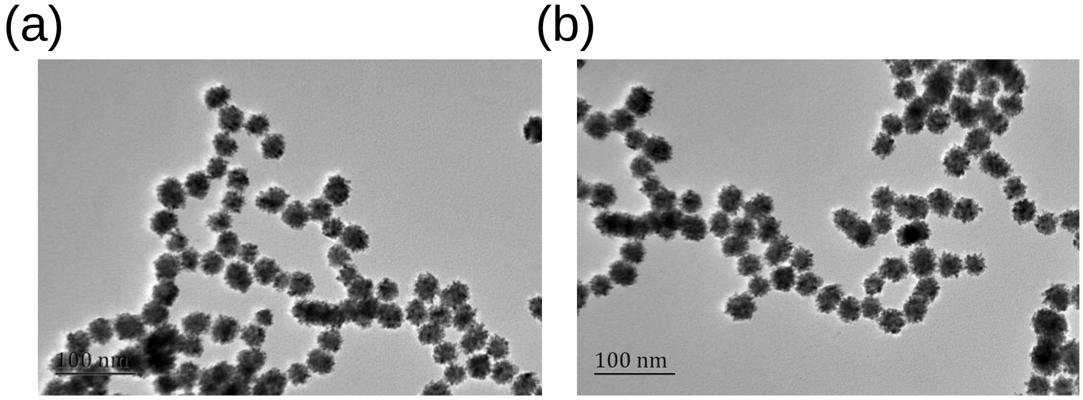

Figure S9. (a) TEM image of Au@Pt NFs (scale ruler is 100 nm); (b) TEM image of antibody-labeled Au@Pt NFs (scale ruler is 100 nm).

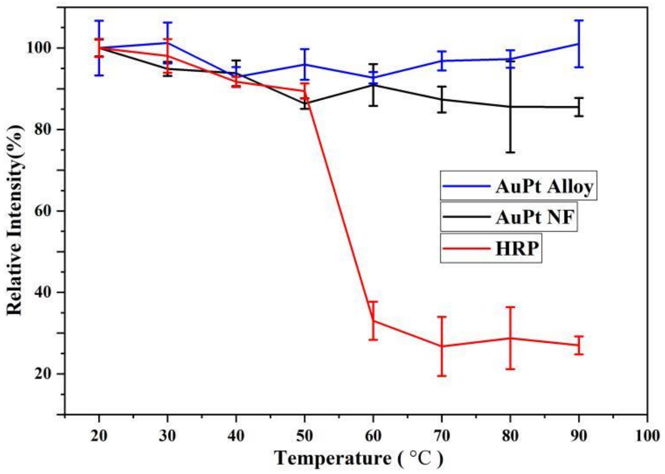

Figure S10. Stability test of three enzymes at different temperatures.

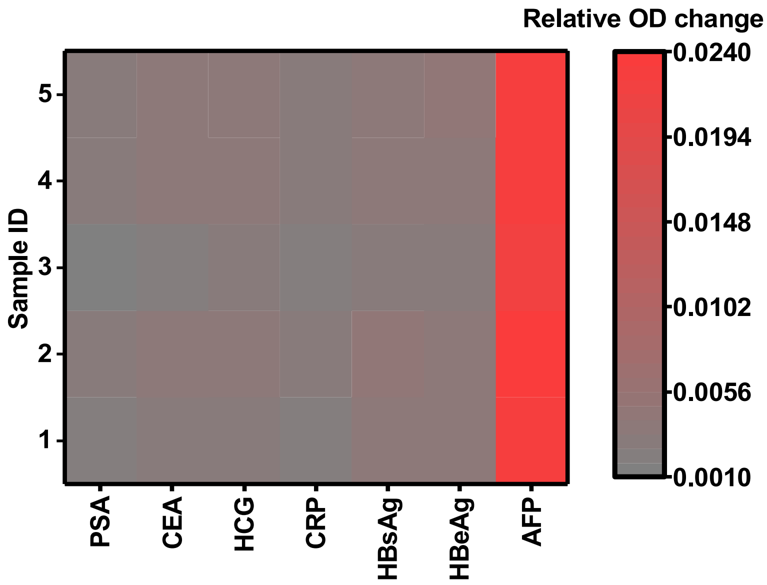

Figure S11. Selectivity of the Nano-ELISPR biosensor for six potential cross-reactant samples.

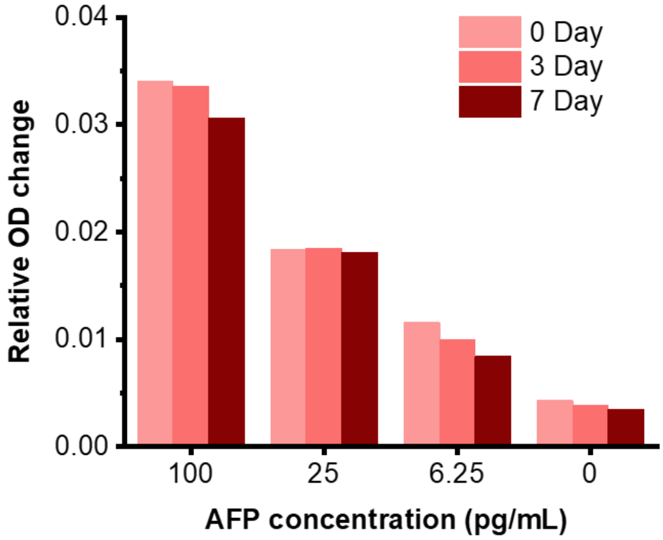

Figure S12. Long-term stability of the Nano-ELISPR platform.

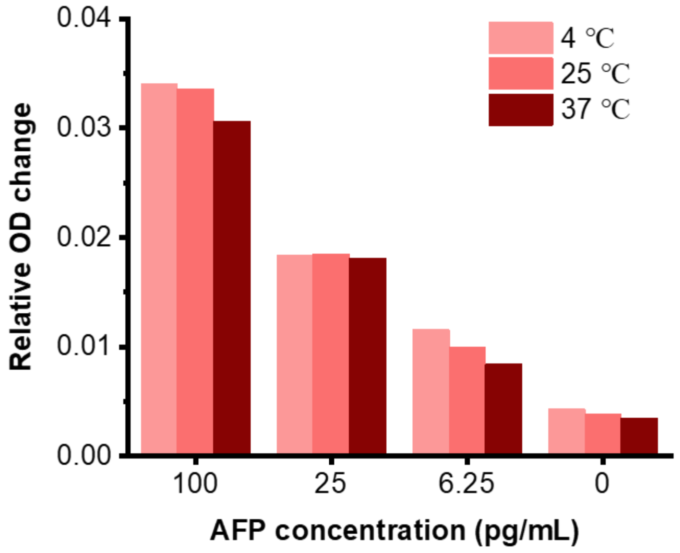

Figure S13. Temperature stability of the Nano-ELISPR platform.
